# Posterior Auricular Mass

**Published:** 2017-06-22

**Authors:** Ian R. Wisecarver, Luke A. Cusimano, Gerhard S. Mundinger

**Affiliations:** ^a^Division of Plastic and Reconstructive Surgery, Louisiana State University, New Orleans, La; ^b^Division of Plastic and Reconstructive Surgery, Children's Hospital of New Orleans, La; ^c^Division of Plastic and Reconstructive Surgery, Tulane University, New Orleans, La

**Keywords:** dermoid, dermoid cyst, auricle, ear mass, soft-tissue mass

## DESCRIPTION

**Figure 1:**
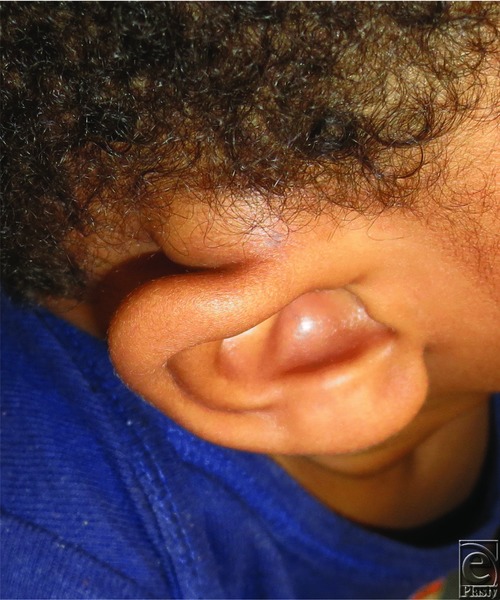
Two-year old with a mass at the posterior/superior right helix at the root.

A 2-year-old African American boy presented with a mass involving the posterior superior right helix at the root (Fig 1). His mother reported that the lesion never caused any symptoms of discomfort and has enlarged slowly since birth. The round, mobile mass was determined to be an isolated finding.

## QUESTIONS

1. **What is differential diagnosis and workup for an auricular mass?**
2. **Where are dermoid cysts most commonly discovered, why do they have a predilection for these areas, and how can they be histologically distinguished from simple epidermal inclusion cysts?**
3. **What is the significance of identifying a dermoid cyst as either midline or peripheral?**
4. **What is the mainstay of treatment?**

## DISCUSSION

The differential diagnosis for masses of the auricle includes dermoid inclusion cyst, epidermoid inclusion cyst, trichilemmal cyst, lipoma, and hemangioma.^1^ Workup of a soft-tissue ear mass is accomplished mainly by physical examination. In the instance of a dermoid cyst, physical examination reveals a firm, rubbery, freely mobile cutaneous soft-tissue mass that may erode bone locally. Ultrasonography may be useful in classifying the mass as cystic, and magnetic resonance imaging can be considered for suspected vascular malformations or intracranial involvement.

The differential diagnosis for masses of the auricle includes dermoid inclusion cyst, epidermoid inclusion cyst, trichilemmal cyst, lipoma, and hemangioma.^1^ Workup of a soft-tissue ear mass is accomplished mainly by physical examination. In the instance of a dermoid cyst, physical examination reveals a firm, rubbery, freely mobile cutaneous soft-tissue mass that may erode bone locally. Ultrasonography may be useful in classifying the mass as cystic, and magnetic resonance imaging can be considered for suspected vascular malformations or intracranial involvement.

Dermoid cysts form embryonically secondary to the inclusion of epidermal and dermal cells between the fusing tissue layers. They are subcutaneous, congenital lesions that most commonly arise at the embryonic fusion lines of facial processes. A large, retrospective case series determined that the upper lateral orbit or orbital rim was the most likely location for a dermoid cyst to occur (80%), followed by the upper medial orbit (10%), and then by the nasal region (5%-10%).^2^ A subsequent analysis of 1495 patients with dermoid cysts, none of whom presented a dermoid cyst of the auricle, corroborates the rarity of auricular dermoid cysts.^3^ Postoperative histological examination of cyst contents confirms the diagnosis and differentiates between dermoid cysts and epidermal inclusions cysts.^1^ The walls of both cyst variants include epidermal layers. Dermoid cysts, however, include dermal layers and adnexal structures.^4^ Dermoid cyst adnexal structures, in the same manner as cutaneous adnexa, produce cellular debris, sebum, and hair. Gradual accumulation of this adnexal waste within the cyst produces the indolent growth that is characteristic of dermoid cysts.^4^

Peripheral dermoid cysts are etiologically and clinically distinct from midline dermoid cysts. The pathologic mechanism of most midline dermoid cysts can be described as persistent fusion of the underlying dura to the overlying ectoderm. As the fetus develops, the dura recedes intracranially, dragging the fused ectodermal tissue with it, forming an inclusion cyst.^5^ The fused dura and ectoderm can remain patent, creating an intracranial connection. The possibility of persistent intracranial connections significantly impacts the course of treatment, and radiologic evaluation becomes crucial to preoperative planning. In contrast, peripheral dermoid cysts are often the result of intrasutural sequestration during calvarial maturation. As such, they are often discovered near suture lines and infrequently present with intracranial involvement.^6^

Surgical excision is the mainstay of treatment of all dermoid cysts. Peripheral dermoid cysts, encompassing 80% of all dermoid lesions, can be excised with minimal preoperative confirmatory imaging. Lesion location and surgeon-specific knowledge of head and neck anatomy allow for regular excision of dermoid cysts, with few, if any, functional or cosmetic consequences. Midline dermoid cysts with intracranial extension are not as easily excised. The formation of a multidisciplinary team, including a neurosurgeon, is the preferred method, as the majority of these cases require a coronal approach with frontal craniotomy for complete cyst excision.^7^

**Figure 2:**
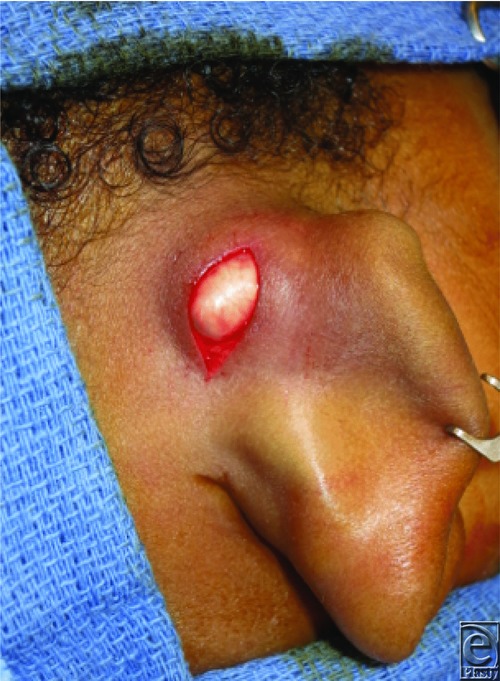
Intraoperative exposure of the postauricular mass.

**Figure 3:**
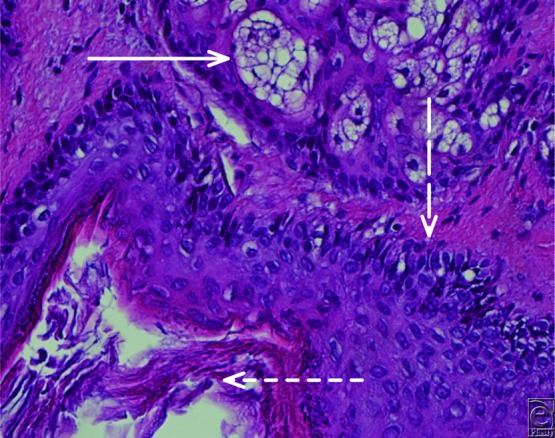
Histological analysis of cyst contents showing adnexal structures (solid arrow), basal layer (dashed arrow), and keratin debris (finely dashed arrow).

**Figure 4:**
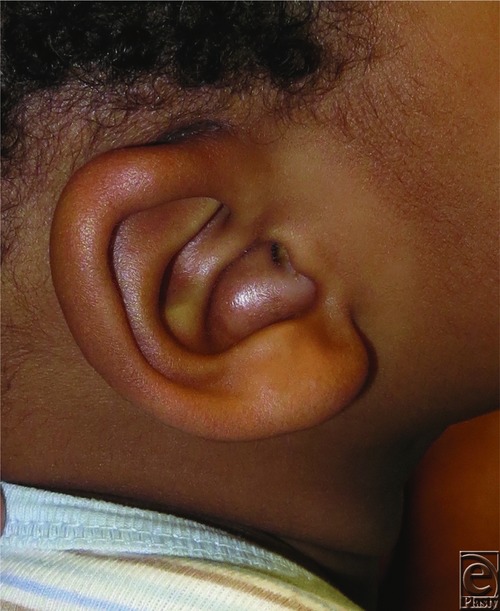
Postoperative follow-up examination showing well-healed incision without evidence of recurrence.

The presented patient was ultimately diagnosed with and treated for postauricular dermoid cyst. Physical examination was highly suggestive of a peripheral dermoid cyst without intracranial involvement, as such there was no preoperative imaging performed. The lesion was completely resected without complications (Fig 2). Postoperative histological analysis demonstrated inclusion of adnexal structures and confirmed the diagnosis (Fig 3). This patient's incision healed well, the mass did not recur, and his overall aesthetic result was excellent (Fig 4).
